# *Annurca* apple polyphenols prevent mercury-induced phosphatidylserine externalization in human erythrocytes via calcium-dependent PLSCR1 regulation

**DOI:** 10.3389/fnut.2026.1770188

**Published:** 2026-02-23

**Authors:** Pasquale Perrone, Claudia Moriello, Nicola Alessio, Alberto Contri, Caterina Manna, Stefania D’Angelo

**Affiliations:** 1 Department of Medical, Human Movement, and Well-Being Sciences (DiSMMeB), Parthenope University of Naples, Naples, Italy; 2 Department of Experimental Medicine, University of Campania “Luigi Vanvitelli”, Naples, Italy; 3 Department of Veterinary Medicine, University of Teramo, Teramo, Italy; 4 Department of Precision Medicine, School of Medicine, University of Campania “Luigi Vanvitelli”, Naples, Italy

**Keywords:** apple, erythrocytes, extracts, flippase, mercury, oxidative stress, phosphatidylserine, scramblase

## Abstract

**Introduction:**

Phosphatidylserine (PS) exposure on the surface of red blood cells (RBC) is a hallmark of membrane asymmetry loss and a prothrombotic signal often induced by oxidative stress and heavy metal toxicity. Mercury (Hg) is known to disrupt cellular redox balance and calcium homeostasis, leading to PS externalization and increased thrombotic risk. Natural antioxidants such as polyphenols may provide protection against these effects. The *Annurca* apple (*Malus pumila* Mill. cv. *Annurca*), a cultivar rich in procyanidins and phenolic compounds, has shown antioxidant and membrane-stabilizing properties.

**Methods:**

Human RBC were isolated from healthy donors and pretreated with *Annurca* apple extracts (0.30 and 1.5 μg catechin equivalents (CAEq)/mL corresponding to 1–5 μM) derived from unripe and ripe fruit (flesh and peel). Cells were then exposed to mercury chloride (HgCl_2_, 20 μM). PS exposure was quantified by annexin V-FITC staining and flow cytometry. Activities and expression levels of ATP11C (flippase) and PLSCR1 (scramblase) were assessed by fluorescence assays, immunocytochemistry, and Western blotting. Intracellular ATP and Ca^2+^ levels were measured using luminescent and fluorometric assays.

**Results:**

HgCl_2_ treatment markedly increased PS exposure, disrupted ATP11C activity, upregulated PLSCR1, elevated intracellular Ca^2+^, and reduced ATP levels. Pretreatment with *Annurca* apple extracts significantly decreased PS exposure in a dose-dependent manner, particularly with ripe fruit extracts. This effect was associated with normalization of intracellular Ca^2+^ and downregulation of PLSCR1, but not with restoration of ATP levels or ATP11C activity. Peel extracts displayed stronger protective effects than flesh extracts.

**Discussion:**

*Annurca* apple polyphenols protect RBC from Hg-induced membrane damage primarily through modulation of calcium-dependent PLSCR1 activity, thereby preserving membrane asymmetry. Unlike other polyphenols such as hydroxytyrosol, *Annurca* extracts act independently of ATP restoration, highlighting a distinct mechanism centered on ionic homeostasis. These findings support the potential of *Annurca* apple as a functional food for mitigating cardiovascular risk associated with heavy metal exposure.

## 1 Introduction

Phosphatidylserine (PS) is an anionic phospholipid essential for the structure and function of cellular membranes. In eukaryotic cells, PS is asymmetrically distributed between the two leaflets of the plasma membrane: under physiological conditions, it is confined almost exclusively to the cytoplasmic leaflet, while it is absent or present only in trace amounts on the extracellular side (1). This asymmetry is crucial for maintaining cellular integrity and for numerous physiological processes, including morphological stability, signal transduction, and the regulation of cell survival (2). The presence of PS in the inner leaflet facilitates the recruitment of cytosolic proteins and the activation of enzymes involved in signaling pathways (3).

Moreover, the externalization of PS to the outer membrane leaflet represents a key physiological signal of programmed cell death (“eat-me signal”) (4). This mechanism is particularly relevant in erythrocytes (RBC), as it enables the selective clearance of senescent or damaged RBC by splenic macrophages, ensuring cellular turnover and maintaining hematological homeostasis (5). However, this phenomenon also has pathological relevance: exposed PS exhibits strong procoagulant properties by providing a negatively charged surface that serves as a platform for prothrombinase complex assembly, promoting thrombin generation and activation of the coagulation cascade (6). Additionally, aberrant PS exposure is associated with the formation of PS-rich RBC-derived microvesicles (MV), further amplifying the prothrombotic potential (7). Increased PS exposure has also been linked to enhanced adhesion of RBC to the endothelium (8). Consequently, non-physiological increases in PS exposure on RBC are closely associated with heightened thrombotic risk and the development of cardiovascular complications through mechanisms including microvascular occlusion and endothelial activation (9).

Maintenance of membrane asymmetry in RBC is regulated by a dynamic enzymatic system that balances active and passive phospholipid transport mechanisms. Specifically, ATP11C, an ATP-dependent P4-ATPase flippase, actively translocates PS from the outer to the inner leaflet of the plasma membrane, thereby preserving lipid asymmetry. ATP11C is the primary flippase in human RBC, as evidenced by the fact that pathogenic mutations in this gene cause congenital hemolytic anemia with up to 90% reduction in flippase activity (10). Loss of ATP11C function has been associated with premature PS exposure and reduced RBC survival (11). In contrast, PLSCR1 (phospholipid scramblase 1) is a Ca^2+^-dependent scramblase that, under oxidative stress or elevated intracellular Ca^2+^, catalyzes bidirectional, non-selective phospholipid translocation, leading to rapid loss of asymmetry (12). The coordinated regulation of ATP11C and PLSCR1 thus represents a central node in modulating PS exposure and RBC physiology (13, 14).

Several studies have shown that pathological conditions such as diabetes mellitus, chronic kidney disease, thalassemia, sickle cell anemia, and, more generally, cardiovascular diseases (CVD) are characterized by increased PS exposure on RBC and the formation of PS-rich MV, contributing to a chronic prothrombotic state (15, 16). These alterations are often accompanied by RBC morphological changes that further impair cellular functionality. Such conditions represent a risk factor for cardiovascular events by promoting hyperactivity of the coagulation cascade, endothelial adhesion, and subsequent vascular damage (17).

Beyond endogenous pathological conditions, exposure to environmental toxic agents, particularly heavy metals, has also been associated with increased PS exposure (18). Mercury (Hg), for example, is known to induce oxidative stress, disrupt redox metabolism, and dysregulate ionic homeostasis in RBC through covalent binding to sulfhydryl groups in glutathione (GSH) and membrane proteins, altering PLSCR1 and ATP11C function and leading to loss of membrane asymmetry (14, 19). These findings highlight how PS accumulation on the RBC surface constitutes a common feature of diverse pathological and toxicological conditions, underscoring the need to identify preventive and therapeutic strategies to mitigate its biological and clinical impact.

In this context, scientific interest is increasingly focused on identifying natural bioactive molecules capable of modulating oxidative stress and preserving cellular function. Our research group has been actively investigating the nutraceutical properties of the *Annurca* apple (*Malus pumila* Miller cv. *Annurca*), a Campanian cultivar particularly rich in polyphenols, proanthocyanidins, and other antioxidant compounds (20, 21). Polyphenolic compounds represent a particularly promising class of natural antioxidants, capable of protecting RBC membranes through mechanisms including direct scavenging of reactive oxygen species (ROS), modulation of endogenous antioxidant systems, and stabilization of membrane structures (22). The *Annurca* apple differs from conventional cultivars due to its significantly higher procyanidin content (up to 20-fold higher than other varieties) and its unique polyphenolic profiles, which confer distinctive nutraceutical properties (23). In previous work, we demonstrated that polyphenolic extracts from *Annurca* apple reduce oxidative stress and improve membrane stability in human RBC, highlighting a potential protective effect (20).

The aim of the present study was to evaluate the protective effect of *Annurca* apple extracts on HgCl_2_-induced PS exposure in human RBC. To this end, RBC were treated with increasing concentrations of *Annurca* apple extracts, and PS exposure was quantified using the annexin V assay. To further investigate the molecular mechanisms underlying the potential protective effect, enzymatic activity of the main regulators of lipid asymmetry, ATP11C and PLSCR1, as well as their membrane expression levels, were assessed. Finally, intracellular calcium and ATP levels, key parameters influencing PLSCR1 and ATP11C activation and the maintenance of membrane asymmetry, were measured to delineate the involved pathophysiological mechanisms.

## 2 Materials and methods

### 2.1 Chemicals and solutions

Phosphate-buffered saline (PBS), bovine serum albumin (BSA) and HgCl_2_ were from Sigma Chemical Co. Nitrobenzoxadiazole-labelled PS (NBD-PS) and Nitrobenzoxadiazole-labelled PC (NBD-PC) were from Avanti Polar Lipids. Annexin V- fluorescein isothiocyanate (V-FITC) Apoptosis Detection Kit (556547, BD Pharmigen, Franklin Lakes, NJ, USA). Bradford reagent and Tris-Glycine gradient gels were from Thermo Fisher Scientific (Waltham, MA, USA). Coomassie Brilliant Blue R250 was from Fluka Chemie (Buchs, Switzerland). Tween-20 was bought from Roche- Diagnostic (Mannheim, Germany). Rabbit anti-ATP11C antibody was from ThermoFisher. Rabbit anti-PLSCR1 antibody was from ElabScience. Goat anti-rabbit IgG (DyLight®_594-conjugated) was from ImmunoReagents. Fluo-3/AM was from Immunotools (Friesoythe, Germany). ATP Assay kit was from Cayman Chemical.

### 2.2 Fruit collection

*Annurca* apples (*Malus pumila* Mill. cv. *Annurca*) were harvested in 2024 from an orchard located in Giugliano in Campania (Naples, Italy). The fruits were harvested in September in the pre-climacteric phase, characterized by green skin and incomplete ripeness. Some of these unripe apples were at once processed for analytical purposes. The remaining fruits underwent the traditional post-harvest reddening process in “melai” which consist of a raised bed of well-drained soil covered with a layer of straw, where the apples were exposed to natural sunlight for about a month. After the reddening phase, samples of ripe fruits were collected and processed for comparative analysis. All experiments were performed using a single harvest batch for unripe apples and a single harvest batch for ripe apples in order to minimize batch-to-batch variability.

### 2.3 Polyphenol extraction

Forty grams of *Annurca* apple sample were homogenized by a Tefal rondo 500 homogenizer using 40 mL of 80% methanol and 20% water plus 0.18 N HCl (15 mL 12 N of HCl/L) for 5 min. After centrifugation (18,000 × *g* for 25 min), the slurry was dried under vacuum by using the Eppendorf Concentrator Plus. The dried extracts were dissolved in 10 mL of PBS and frozen at −80 °C until use. The total polyphenolic content of apple extracts was estimated using the Folin–Ciocalteu phenolic reagent. The extracts (100 μL) were mixed with Folin–Ciocalteu phenolic reagent (0.5 mL), deionized water (0.9 mL), and Na_2_CO_3_ (7.5% w/v, 4 mL). The absorbance at 765 nm was measured 2 h after incubation at room temperature using a UV-3100PC spectrophotometer. The measurement was compared to a standard curve of prepared catechin solutions and expressed in milligrams of catechin equivalent (CAEq) per 100 g FW (fresh weight) of apple sample. To ensure consistency and comparability between studies, the extracts used in this work originate from the same batches previously prepared and characterized in a recent manuscript from our group. These extracts were produced using the identical extraction protocol, stored under the same experimental conditions, and their polyphenolic composition was characterized by HPLC chromatographic analysis, allowing the identification and quantification of the main phenolic constituents (21).

### 2.4 Preparation of red blood cells and treatment with HgCl_2_

Whole blood was obtained with informed consent from healthy volunteers at the University of Campania “Luigi Vanvitelli” (Naples, Italy). It was collected in heparinised tubes and centrifuged at 2000 × *g* for 10 min at 4 °C. The buffy coat was then removed, and the RBC fraction was washed three times with isotonic saline solution (0.9% NaCl) and resuspended in Krebs solution containing (mM) NaCl 125, KCl 4, MgSO_4_ 1, Hepes 32, CaCl_2_ 1, glucose 5; pH 7.4 to obtain a different haematocrit as required. RBC were pretreated with different concentrations of apple extracts (0.30–1.5 μg CAEq/mL) for 15 min and then incubated for 4 h at 37 °C with HgCl_2_ (20 μM). The concentration of HgCl_2_ was selected to induce pronounced oxidative and ionic stress in erythrocytes while preserving cell integrity, thus allowing the analysis of early membrane alterations rather than nonspecific hemolysis.

### 2.5 Detection of annexin-V-binding cells

After incubation under the respective conditions, RBC (haematocrit 0.4%) were washed three times in Krebs solution. They were then resuspended in 500 μL of 1 × binding buffer with 5 μL of Annexin-V apoptosis detection kit. They were then incubated in the dark for 15 min at room temperature. Fluorescence assessment was performed with BD AccuriC6, data were analysed on the FACS Calibur flow cytometer and evaluated with FlowJo V10 software.

### 2.6 Measurement of flippase and scramblase activities

Enzyme activity was measured following the protocol described by Seki et al. (11). In brief, after the respective treatments, RBC (haematocrit 2%) were incubated at 4 °C for 30 min with 2.5 μM of NBD-PS for the flippase activity test or NBD-PC for the scramblase activity test. Subsequently, the cells were mixed with PBS in the presence or absence of 5% BSA to remove residual NBD-PS/PC in the outer sheet. In this way, it can be demonstrated that the residual fluorescence associated with the cells represents the PS/PC translocated to the inner sheet. The amount of internalized probe was calculated by dividing the fluorescence intensity associated with red blood cells before and after extraction with BSA. The samples were analysed on the FACS Calibur flow cytometer and evaluated with FlowJo V10 software.

### 2.7 Measurement of intracellular ATP levels

The intracellular ATP level was measured using the ATP Assay kit according to the manufacturer’s instructions. Briefly, after the respective incubation conditions, RBC (haematocrit 0.4%) were washed with cold PBS and lysed with cold 1X ATP sample buffer. Then, 1 μL of each sample was placed in a 96-well plate and subsequently 100 μL of freshly prepared reaction mixture (1× ATP detection buffer, D-Luciferin and Luciferase) was added. The plate was then incubated at room temperature for 20 min, protected from light. The luminescence intensity was detected by the Spark 10 M multimodal microplate reader (TECAN) and the ATP concentration was calculated according to the manufacturer’s instructions.

### 2.8 Measurement of intracellular calcium levels

Intracellular calcium levels were measured using the Fluo-3 probe. After incubation, 100 μL of RBC suspension (haematocrit 2%) was washed in PBS and treated with 5 μM Fluo-3/AM. The cells were incubated at 37 °C for 30 min. Subsequently, the Fluo-3/AM-loaded RBC were washed and resuspended in 200 μL of PBS. Fluorescence intensity was measured at an excitation wavelength of 488 nm and an emission wavelength of 530 nm on a FACSCalibur flow cytometer and evaluated using FlowJo V10 software.

### 2.9 Immunocytochemistry

After the respective treatments, RBC (haematocrit 1%) were washed twice in PBS. They were then fixed with 300 μL of 1% formaldehyde and incubated for 10 min at room temperature. After two further washes in PBS, 300 μL of blocking solution (5% FBS) was added to the cells and incubated for 30 min at room temperature. The cells were then washed twice in PBS and treated with the primary antibodies Anti-glycoprotein A (1:200), Anti-PLSCR1 (1:200) and Anti-ATP11C (1:200) and incubated for 1 h at room temperature in the dark. After two further washes in PBS, the solution containing the secondary antibody Anti-rabbit (1:400) was added. After incubating the cells for 45 min in the dark at room temperature, 50 μL of cell solution was taken, placed on coverslips and left at room temperature overnight. The following day, the samples were viewed under a ZEISS Axioscope microscope.

### 2.10 Preparation of RBC membranes and protein extraction

After treatment with HgCl_2_, RBC (haematocrit 10%) were washed twice with PBS and centrifuged at 2000 g for 10 min at 4 °C. The samples were incubated for 1 h at 4 °C with a hypotonic 0.1× PBS solution under agitation to achieve cell lysis. Subsequently, the membranes were separated from the intracellular content by ultracentrifugation at 21,500 g, 75 min, 4 °C. The supernatant, containing the cytosolic fraction, was separated from the bottom fraction containing the membranes. The membranes were then washed eight times by centrifugation at 21,500 g for 30 min at 4 °C to remove as much hemoglobin as possible from the membranes. Total membrane proteins were then extracted from the pelleted membranes under native conditions using DC buffer: 1% DC in 50 mM Tris–HCl, 150 mM NaCl, pH 8.1. A final centrifugation was performed at 21,500 g, 30 min, 4 °C. Protein concentrations were measured by absorbance using Bradford reagent and a Cary ultraviolet–visible spectrophotometer.

### 2.11 Western blotting

Total membrane protein extracts were analysed by SDS-PAGE. 40 μg of protein from each sample with the addition of 10 μL of 1× Laemmli buffer were boiled at 100 °C for 10 min and then loaded onto a 1.0 mm 4%–8% Tris-Glycine gradient gel. Electrophoresis was performed in 2× Tris-Glycine running buffer at 150 V for approximately 2 h. SDS-PAGE was performed as a preparative step for subsequent Western blot analysis.

Western blotting was performed on nitrocellulose membranes (1 h transfer at 10 V in Tris-glycine buffer in a Mini Trans-Blot electrophoretic transfer cell). The membranes were then rinsed three times for 10 min in TBS-T (1× TBS, 0.1% Tween-20) and blocked with 5% skim milk in TBS-T for 1 h at room temperature with agitation. They were then incubated separately with the following primary monoclonal antibodies in TBS-T: rabbit anti-ATP11C antibody (1:500), rabbit anti-PLSCR1 antibody (1:1000) and rabbit anti-*β* actin antibody (1:4000). After overnight incubation, the membranes were rinsed three times for 10 min in TBS-T. The membranes were then incubated with secondary antibodies (HRP-conjugated polyclonal rabbit anti-mouse immunoglobulin) (HRP-conjugated polyclonal goat anti-rabbit immunoglobulin) diluted 1:2000 and 1:4000, respectively, in 3% skim milk buffer in TBS-T for 2 h at room temperature with agitation. Finally, the membranes were washed three times in TBS-T at room temperature. The HRP reaction was detected using a chemiluminescence kit (ECL Western Blotting Substrate, Pierce, Waltham, MA, USA) and images were acquired using the ChemiDoc system (Bio-Rad, Hercules, CA, USA). The immunopositive signal corresponding to the enzymes was quantified by densitometric analysis using Image Lab 6.0.1 software. The levels of ATP11C and PLSCR1 were compared with the intensity of GAPDH.

### 2.12 Statistical analyses

Data evaluations were expressed as means ± S.D. of 3 independent experiments performed in triplicate with RBC from different donors. The significance of differences was determined by one-way ANOVA followed by a post Tukey’s multiple comparisons test. GraphPad Prism 10 was utilized for statistical analysis.

## 3 Results

### 3.1 Phosphatidylserine exposure analysis

**Figure 1:**
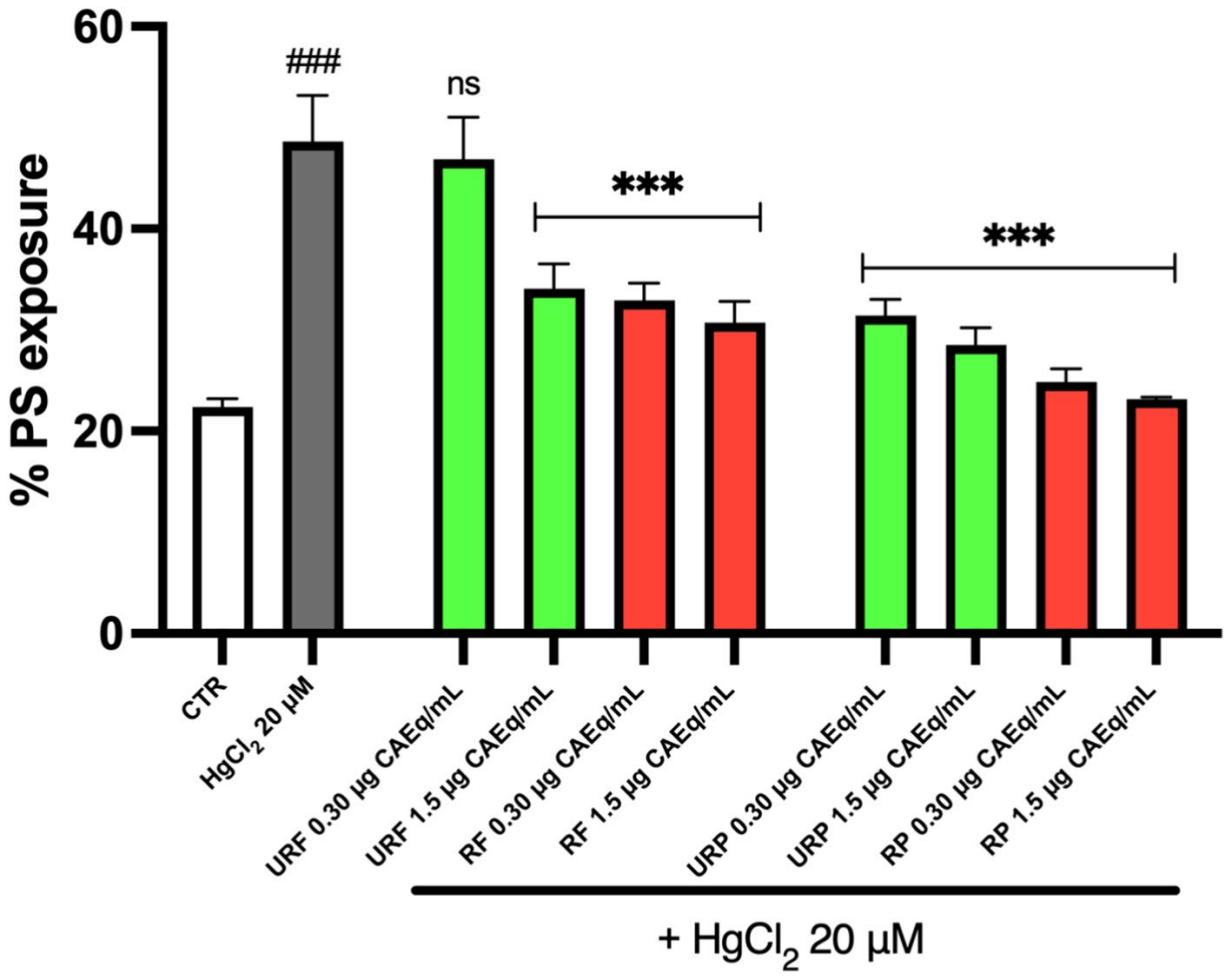
Effect of different *Annurca* apple extracts on Hg-induced PS exposure in RBC. Cells were treated with HgCl_2_ in the presence of increasing concentrations of unripe flesh, ripe flesh, unripe peel, and ripe peel. Data are the means ± SD (*n* = 3). Statistical analysis was performed with ANOVA followed by Tukey’s test. ###(*p* < 0.001) indicates a significant difference from CTR. ***(*p* < 0.001) indicates a significant difference fromHgCl_2_ treatment. ns indicates no significant difference from HgCl_2_ treatment.

The first step of the study was to evaluate whether *Annurca* apple extracts could modulate Hg-induced PS exposure. As shown in Figure 1, Hg treatment caused a significant increase in PS exposure, approximately 2.5-fold higher than in the control. However, the data indicate that *Annurca* apple extracts are able to partially counteract this effect.

Specifically, extracts from unripe flesh (URF) at low concentrations (0.30 μg CAEq/mL) did not exhibit significant protective effects. In contrast, both URF extracts at higher concentration (1.5 μg CAEq/mL) and ripe flesh extracts (RF) at both tested concentrations (0.30 and 1.5 μg CAEq/mL) displayed a significant protective effect. A similar pattern was observed for peel extracts, both unripe (URP) and ripe (RP), with evidence of a dose-dependent effect; higher concentrations (1.5 μg CAEq/mL) resulted in a greater reduction of PS exposure.

Moreover, ripe apple extracts, both from flesh and peel, demonstrated a generally stronger protective effect compared to the corresponding extracts from unripe apples.

### 3.2 Measurement of flippase and scramblase activities

To assess the enzymatic activity of the proteins responsible for PS translocation, flippase ATP11C and scramblase PLSCR1, a fluorescence-based assay employing NBD-PS and NBD-PC analogues was performed.

**Figure 2:**
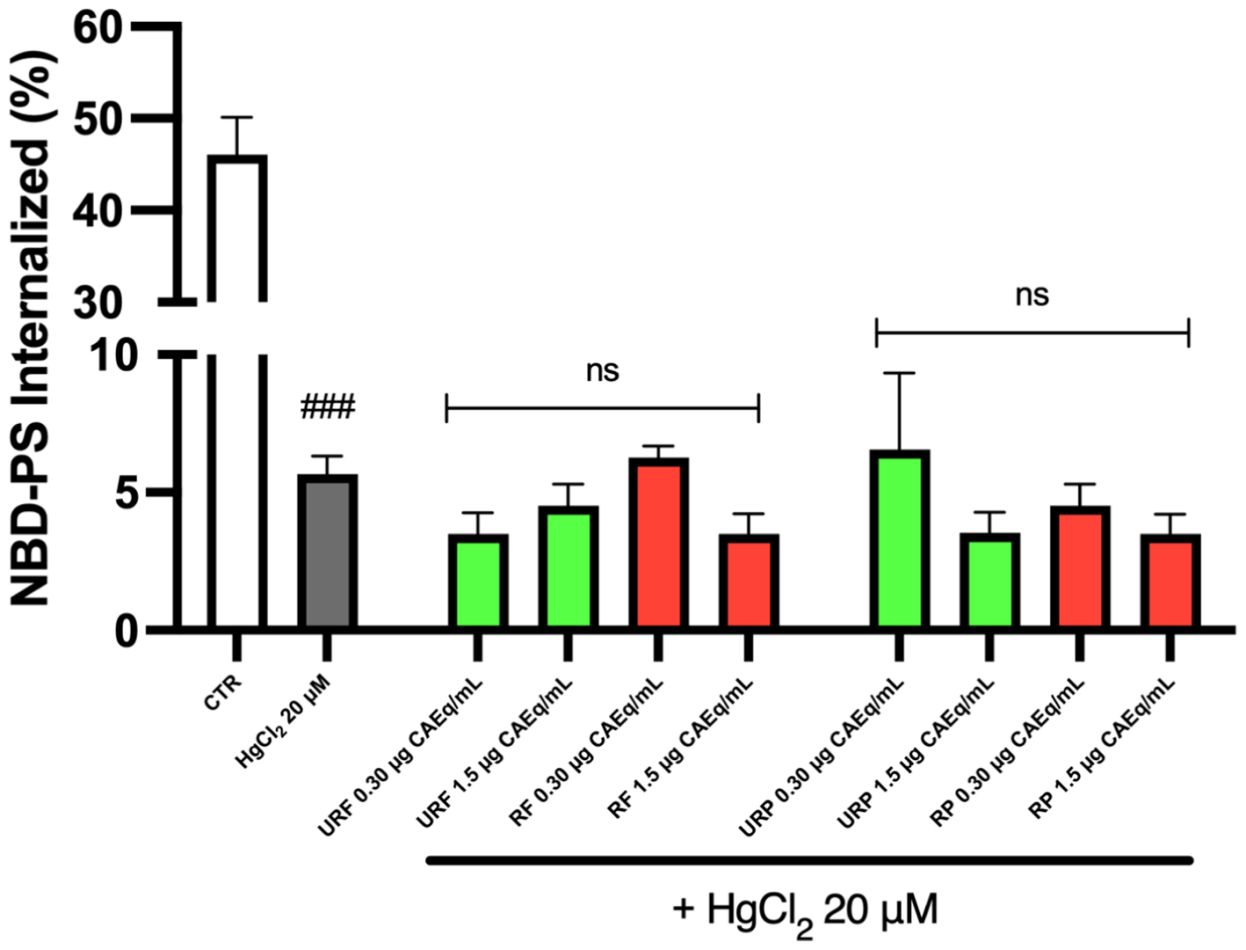
Effect of different *Annurca* apple extracts on Hg-induced NBD-PS internalization in RBC. Cells were treated with HgCl_2_ in the presence of increasing concentrations of unripe flesh, ripe flesh, unripe peel, and ripe peel. Data are the means ± SD (*n* = 3). Statistical analysis was performed with ANOVA followed by Tukey’s test. ###(*p* < 0.001) indicates a significant difference from CTR. ns indicates no significant difference from HgCl_2_ treatment.

As shown in Figure 2, the amount of internalized NBD-PS markedly decreased (approximately tenfold) following Hg treatment, indicating a substantial impairment of ATP11C enzymatic activity. Notably, pretreatment with *Annurca* apple extracts failed to reverse the toxic effects of the heavy metal, regardless of the fruit’s ripening stage or the type of extract (flesh or peel).

As previously reported, PS transport is mediated by both flippase and scramblase. Therefore, scramblase activity has also been assessed. To discriminate the activity of these two enzymes, the fluorescent analogue of PC, NBD-PC, was used, as this phospholipid is solely transported by scramblase.

**Figure 3:**
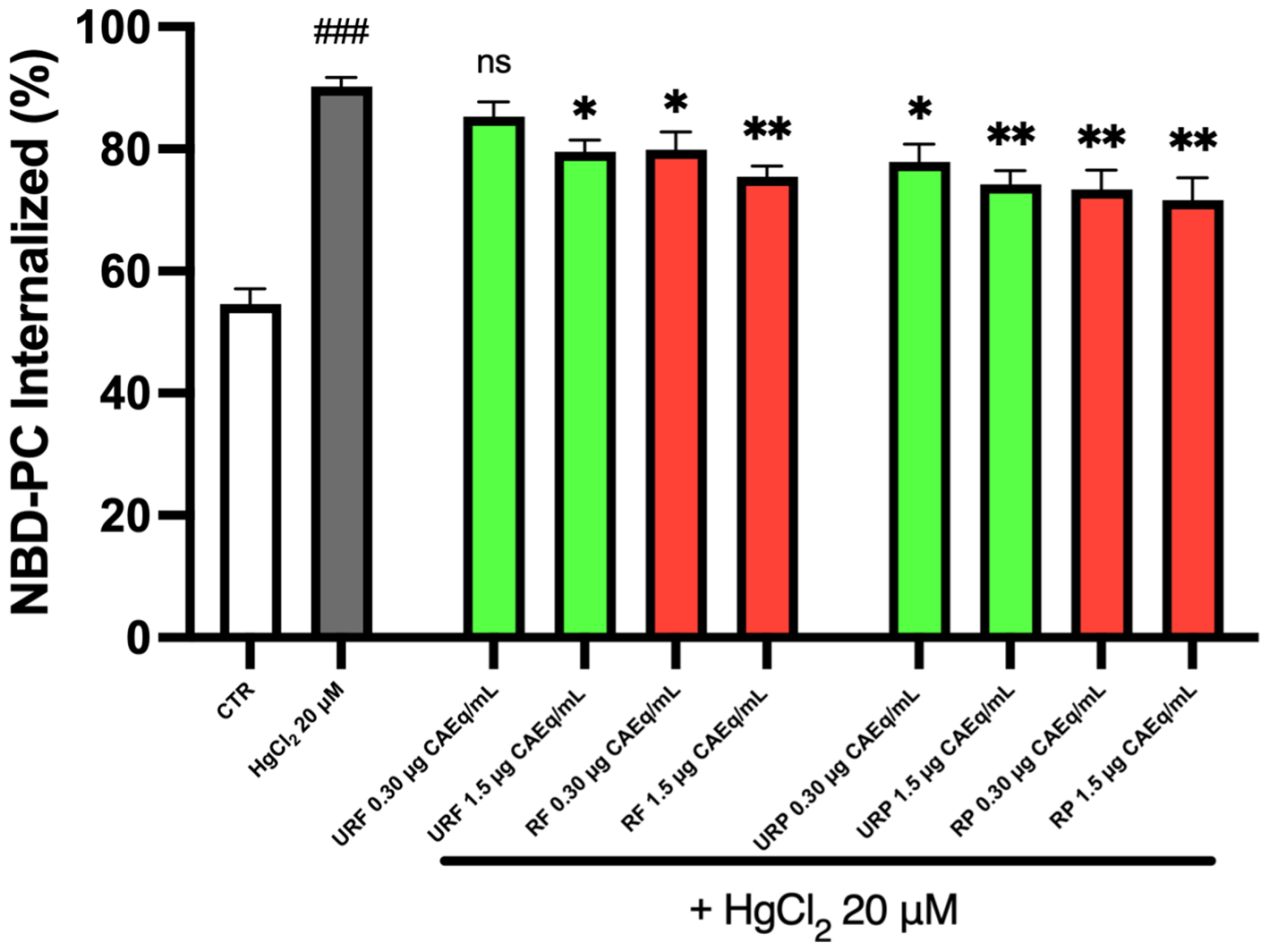
Effect of different *Annurca* apple extracts on Hg-induced NBD-PC internalization in RBC. Cells were treated with HgCl_2_ in the presence of increasing concentrations of unripe flesh, ripe flesh, unripe peel, and ripe peel. Data are the means ± SD (*n* = 3). Statistical analysis was performed with ANOVA followed by Tukey’s test. ###(*p* < 0.001) indicates a significant difference from CTR. **(*p* < 0.01) and *(*p* < 0.05) indicate a significant difference from HgCl_2_ treatment. ns indicates no significant difference from HgCl_2_ treatment.

The data presented in Figure 3 indicate that, once again, Hg treatment led to an alteration of the physiological enzymatic activity. Specifically, since PC is normally localized on the outer leaflet of the plasma membrane, an increased exposure of PC can be interpreted as a marker of enzymatic dysfunction. However, the data demonstrate that pretreatment with *Annurca* apple extracts reduces PC exposure on the membrane. Notably, only the unripe flesh (URF) sample at low concentration (0.3 μg CAEq/mL) did not show a protective effect, whereas such protection was observed in both ripe and unripe flesh samples and in all peel extracts.

### 3.3 Measurement of intracellular ATP and calcium levels

As aforementioned, ATP11C is an ATP-dependent enzyme while PLSCR1 is a Ca^2+^-dependent enzyme, thus, intracellular ATP and Ca^2+^ levels were assessed, to determine the possible mechanisms of action for effects described above (13).

**Figure 4:**
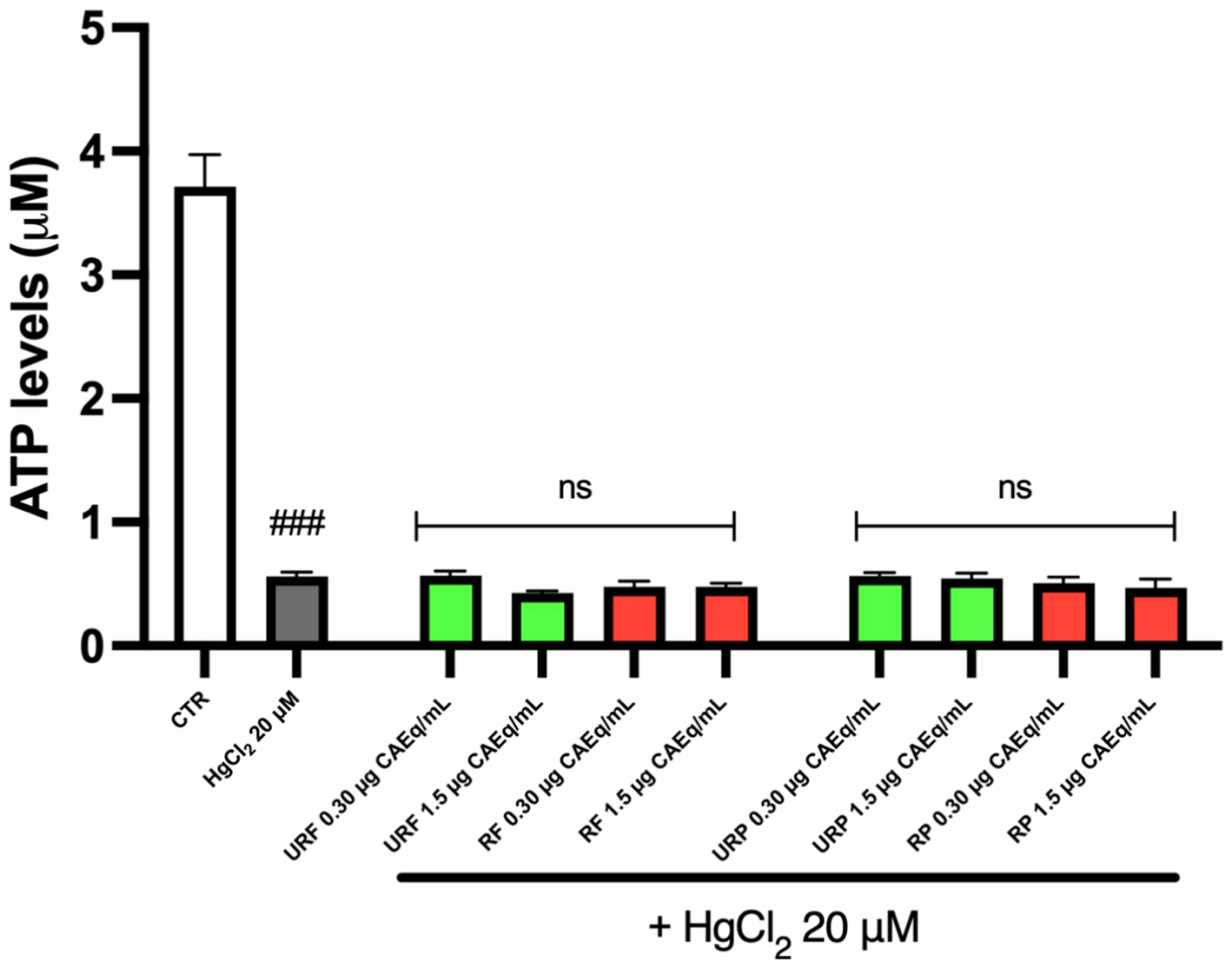
Effect of different *Annurca* apple extracts on Hg-induced ATP intracellular levels alterations in RBC. Cells were treated with HgCl_2_ in the presence of increasing concentrations of unripe flesh, ripe flesh, unripe peel, and ripe peel. Data are the means ± SD (*n* = 3). Statistical analysis was performed with ANOVA followed by Tukey’s test. ###(*p* < 0.001) indicates a significant difference from CTR. ns indicates no significant difference from HgCl_2_ treatment.

As shown in Figure 4, intracellular ATP levels were significantly reduced following the treatment of RBC with the heavy metal. However, the data indicate that treatment with *Annurca* apple extracts was not able to restore physiological ATP levels, regardless of the fruit’s ripening stage or the type of extract used.

**Figure 5:**
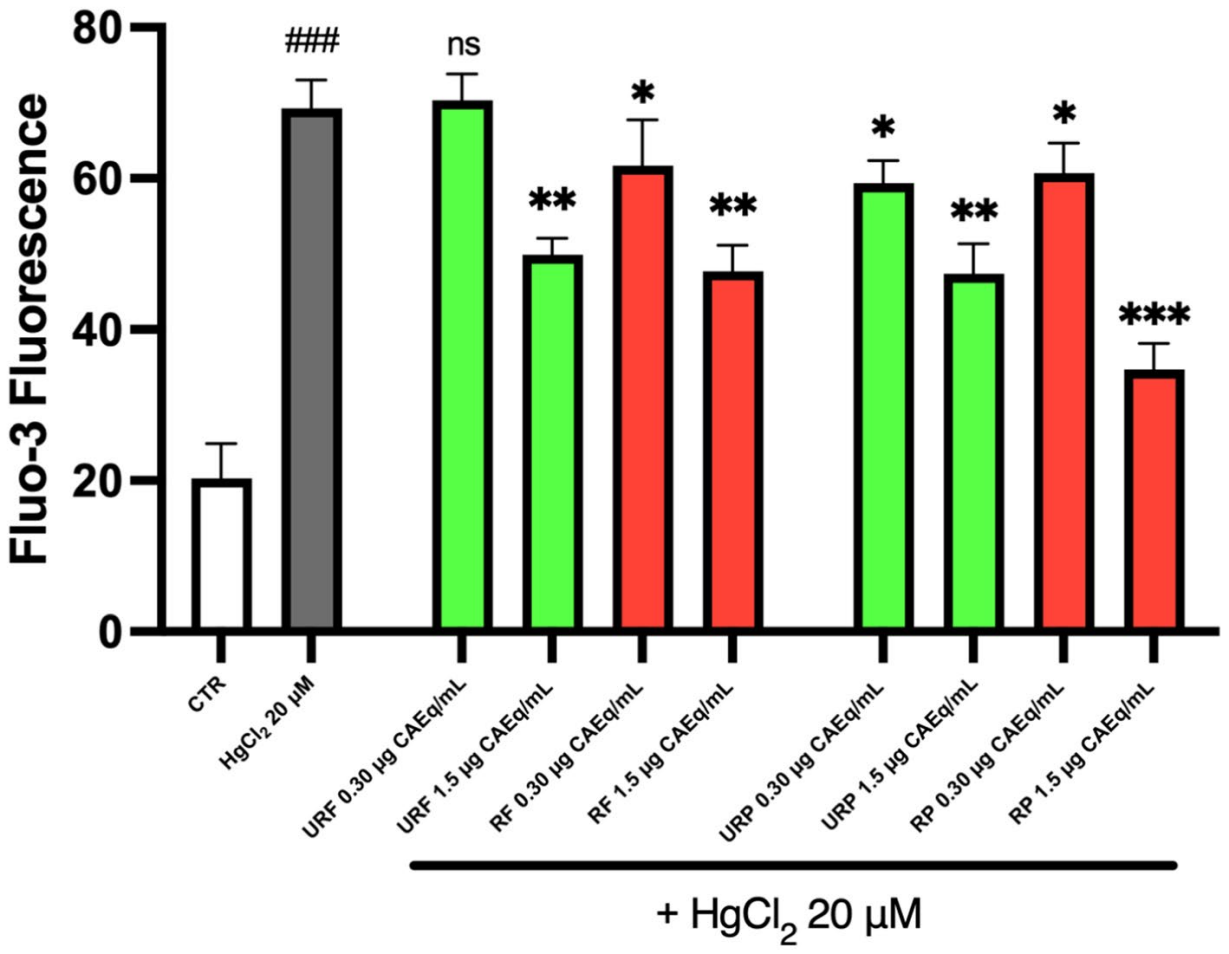
Effect of different *Annurca* apple extracts on Hg-induced Ca^2+^ intracellular levels alterations in RBC. Cells were treated with HgCl_2_ in the presence of increasing concentrations of unripe flesh, ripe flesh, unripe peel, and ripe peel. Data are the means ± SD (*n* = 3). Statistical analysis was performed with ANOVA followed by Tukey’s test. ###(*p* < 0.001) indicates a significant difference from CTR. ***(*p* < 0.001), **(*p* < 0.001) and *(*p* < 0.05) indicate a significant difference from HgCl_2_ treatment. ns indicates no significant difference from HgCl_2_ treatment.

As shown in Figure 5, the data illustrate the variations in intracellular Ca^2+^ concentration. Treatment with Hg resulted in an approximately 3.5-fold increase in intracellular calcium levels compared to the control. However, pretreatment with *Annurca* apple extracts markedly attenuated this toxic effect. In particular, the extracts URF 1.5 μg CAEq/mL, RF 1.5 μg CAEq/mL, and URP 1.5 μg CAEq/mL exhibited comparable protective effects. Similarly, samples treated with RF 0.3 μg CAEq/mL, URP 0.3 μg CAEq/mL, and RP 0.3 μg CAEq/mL showed a similar reduction in calcium accumulation. Interestingly, only the URF 0.3 μg CAEq/mL sample did not show statistical significance, indicating the absence of a relevant protective effect.

### 3.4 Immunocytochemistry

The enzymatic activity analyses of ATP11C and PLSCR1 were further supported by the evaluation of their plasma membrane expression.

**Figure 6:**
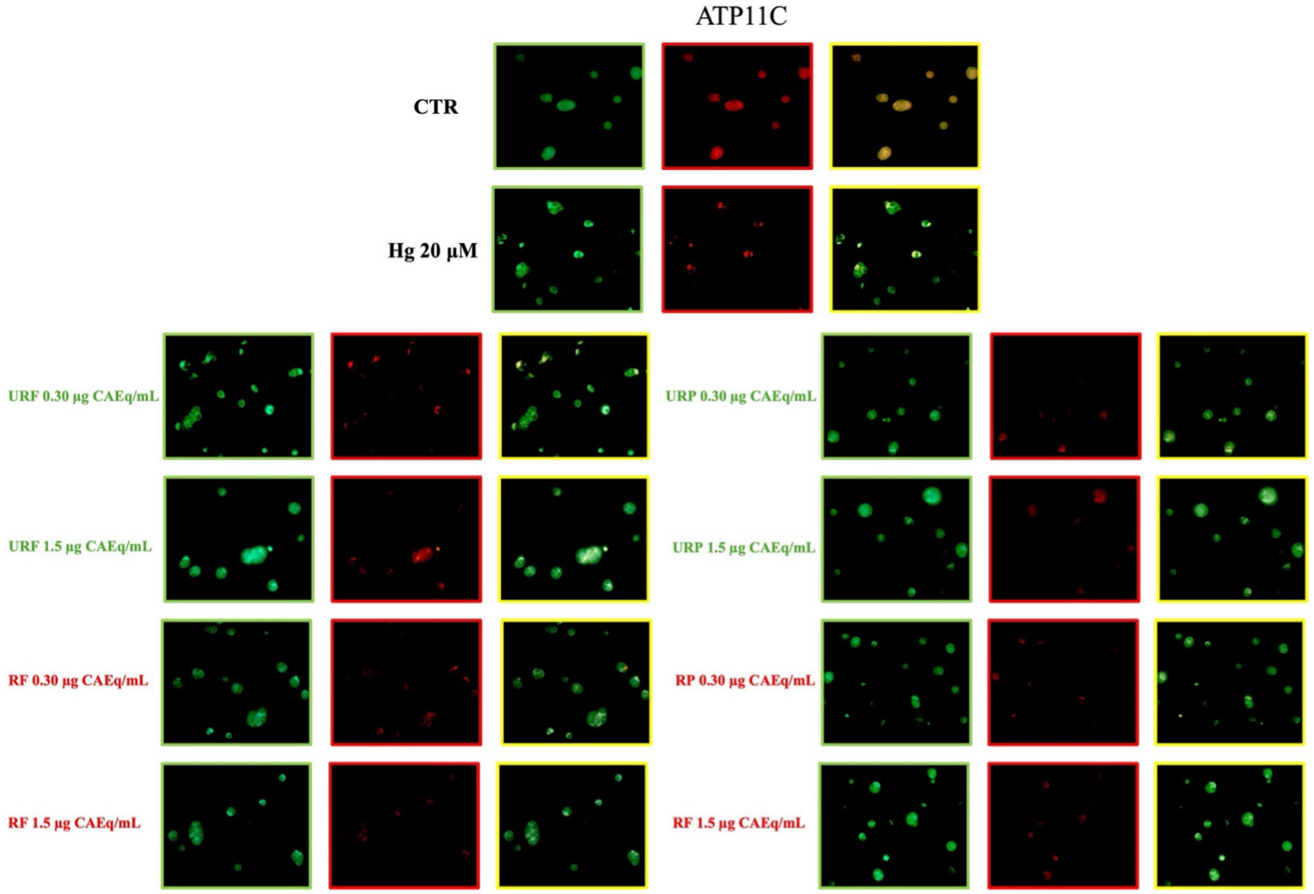
Effect of different *Annurca* apple extracts on Hg-induced ATP11C expression on RBC membrane. Cells were treated with HgCl_2_ in the presence of increasing concentrations of unripe flesh, ripe flesh, unripe peel, and ripe peel. Flippase expression was analysed using immunocytochemistry. Green: Glycophorin-A; Red: ATP11C; Yellow: merge.

As shown in Figure 6, immunocytochemical analysis of ATP11C revealed that treatment with Hg caused a substantial reduction in the membrane expression of this enzyme. The MERGE signal (in yellow) and the specific ATP11C signal (in red) were markedly lower than those observed in the control samples. Interestingly, in this case as well, treatment with *Annurca* apple extracts did not appear to exert any significant effect, as the signal intensity did not increase regardless of the type of extract used.

**Figure 7:**
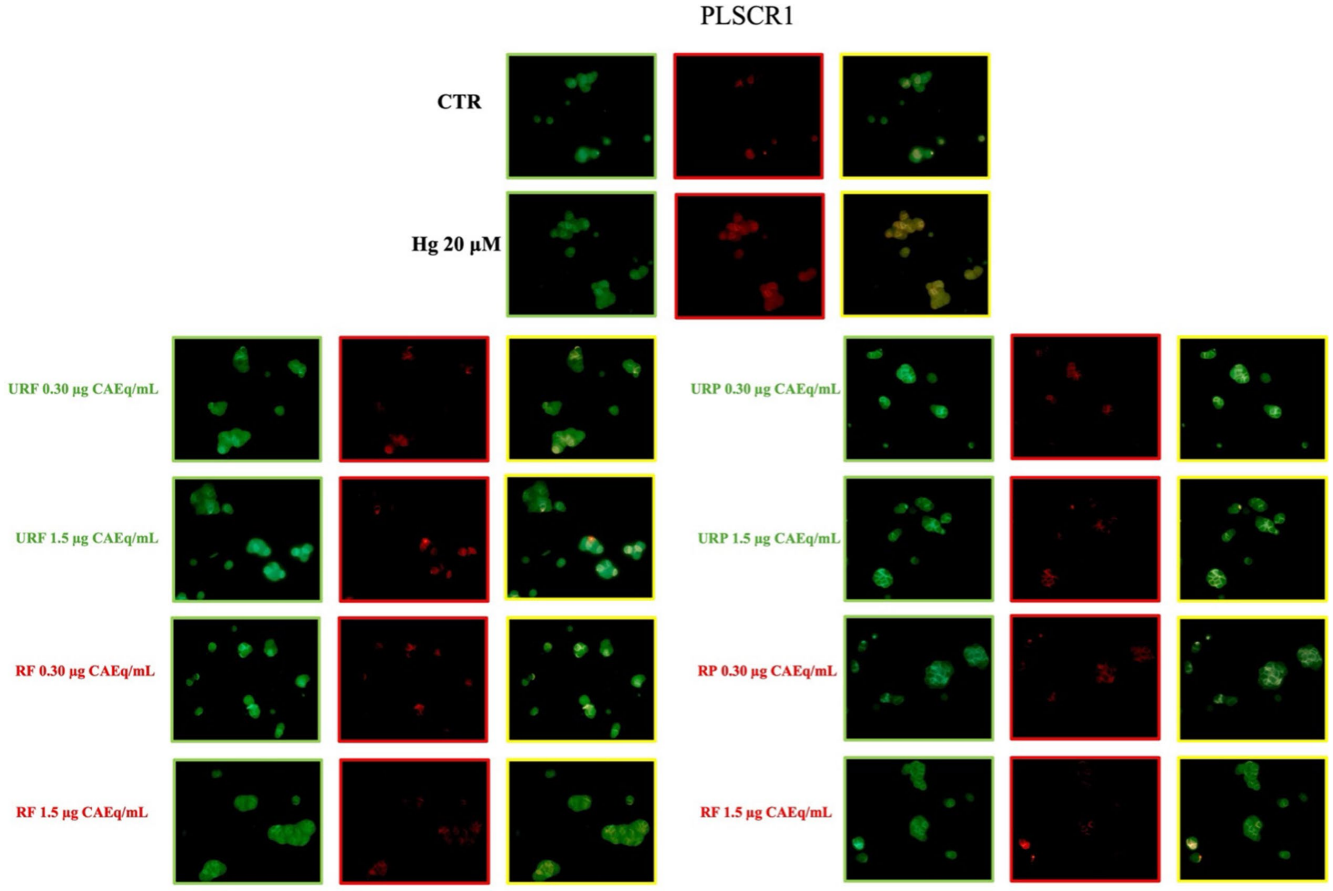
Effect of different *Annurca* apple extracts on Hg-induced PLSCR1 expression on RBC membrane. Cells were treated with HgCl_2_ in the presence of increasing concentrations of unripe flesh, ripe flesh, unripe peel, and ripe peel. Flippase expression was analysed using immunocytochemistry. Green: lycophorin-A; Red: ATP11C; Yellow: merge.

A markedly different pattern was observed for the enzyme PLSCR1. As shown in Figure 7, treatment with Hg also caused a pronounced alteration in the membrane expression of this enzyme. Specifically, a significant increase in signal intensity was detected, indicating a higher expression level compared to the control. Interestingly, treatment with *Annurca* apple extracts markedly reduced the membrane expression of PLSCR1, restoring it to levels comparable to the physiological condition.

### 3.5 Western blotting

**Figure 8:**
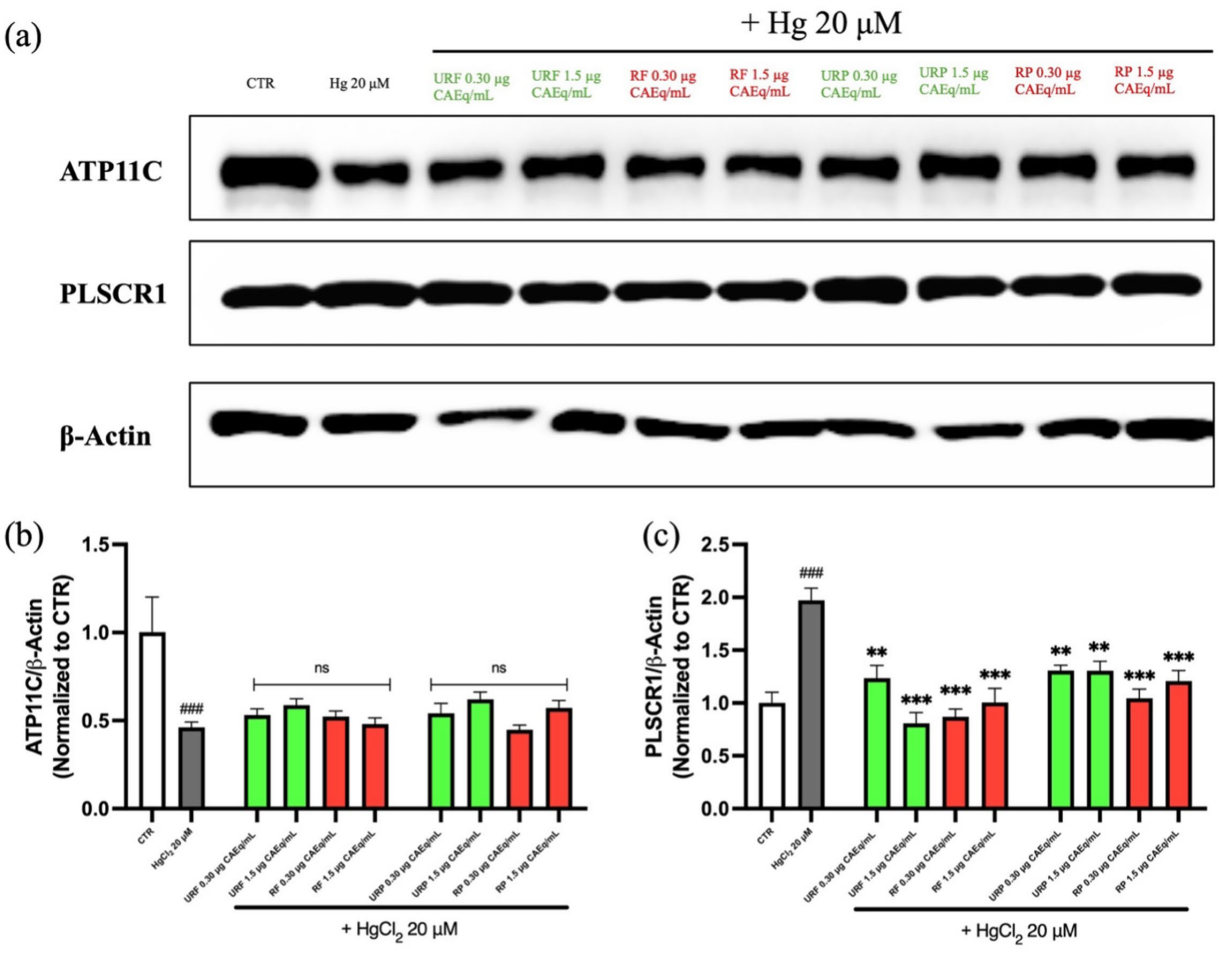
Effect of different *Annurca* apple extracts on Hg-induced ATP11C and PLSCR1 expressions on RBC membrane. Cells were treated with HgCl_2_ in the presence of increasing concentrations of unripe flesh, ripe flesh, unripe peel, and ripe peel. **(a)** Western blot analysis. **(b)** Densiometric analysis of ATP11C expression. **(c)** Densiometric analysis of PLSCR1 expression. Data are the means ± SD (*n* = 3). Statistical analysis was performed with ANOVA followed by Tukey’s test. ###(*p* < 0.001) indicates a significant difference from CTR. ***(*p* < 0.001) and **(*p* < 0.001) indicate a significant difference from HgCl_2_ treatment. ns indicates no significant difference from HgCl_2_ treatment.

The results obtained from the immunocytochemical analysis were further confirmed by Western blotting. As shown in Figure 8, the expression levels of the enzymes are consistent with the findings described above. Specifically, for the enzyme ATP11C, a ~50% decrease in band intensity was observed in the samples treated with the heavy metal. In agreement with previous data, samples also treated with *Annurca* apple extracts, regardless of the extract type, did not show any protective effect or restoration of physiological expression levels.

Conversely, for the enzyme PLSCR1, a twofold increase in band intensity was detected in the Hg-treated samples. However, consistent with the previous observations, treatment with *Annurca* apple extracts markedly reduced the band intensity, restoring it to levels comparable to the control, thus indicating a protective effect.

## 4 Discussion

Cardiovascular diseases (CVD) remain the leading cause of death in industrialized countries, accounting for a major burden of morbidity and mortality worldwide (24). The underlying pathophysiological mechanisms are multifactorial and complex, involving oxidative stress, chronic inflammation, endothelial dysfunction, and hyperactivation of the coagulation cascade (25, 26). Among emerging risk factors, exposure to heavy metals, particularly Hg, represents an increasingly recognized environmental threat to cardiovascular health (27). Hg is widely distributed in the environment through waste incineration, industrial activities, and contamination of water resources, resulting in human exposure mainly through the consumption of contaminated food, especially fish and seafood (28). From an epidemiological perspective, several prospective and cross-sectional studies have documented a significant association between blood Hg levels and increased risk of hypertension, coronary artery disease, atherosclerosis, and acute myocardial infarction (29).

In this context, the identification of natural bioactive compounds capable of counteracting heavy metal-induced toxicity has become a major scientific and clinical objective (30, 31). The present study provides experimental evidence of the efficacy of polyphenolic compounds extracted from the *Annurca* apple in preventing metabolic and morphological damage in human RBC exposed *in vitro* to HgCl_2_, representing an innovative nutritional approach to mitigating cardiovascular toxicity associated with heavy metal exposure.

The most relevant finding of this study is the ability of *Annurca* apple extracts to significantly and dose-dependently reduce Hg-induced PS exposure on RBC surface. PS externalization, a hallmark of oxidative damage and a potent procoagulant signal, was markedly attenuated by pretreatment with the extracts, particularly those derived from ripe fruits, both flesh and peel. Extracts from unripe fruits exhibited a weaker effect, suggesting that the ripening process, which is associated with an increased concentration of active polyphenols, enhances the protective capacity of *Annurca* (21). This finding has important pathophysiological implications since PS exposed on the RBC membrane provides a negatively charged surface that serves as a platform for the assembly of the prothrombinase complex, promoting thrombin generation and coagulation cascade activation (32). The reduction in PS exposure observed in the presence of *Annurca* extracts therefore suggests a potential antithrombotic and vasculoprotective effect.

Analysis of the underlying molecular mechanisms revealed that the protective action of *Annurca* extracts is primarily associated with the normalization of intracellular calcium levels and the consequent modulation of the scramblase PLSCR1. Treatment with Hg caused an approximately 3.5-fold increase in intracellular Ca^2+^ concentration, an event that triggers PLSCR1 activation and loss of membrane lipid asymmetry. The mechanism through which Hg induces intracellular Ca^2+^ accumulation is multifaceted: covalent binding of Hg to sulfhydryl groups of glutathione and membrane proteins may impair calcium chelation systems and directly interfere with ion channels and Ca^2+^-ATPase pumps (33). Pretreatment with *Annurca* extracts markedly reduced this calcium overload, suggesting that the polyphenolic mixture acts by preventing Hg-induced ionic dysregulation. The dose-dependent effect, more pronounced at 1.5 μg CAEq/mL, and the higher efficacy of ripe fruit extracts indicate a direct correlation between total polyphenol content and the ability to modulate calcium homeostasis.

The mechanism by which *Annurca* extracts counteract calcium accumulation likely involves multiple convergent pathways. For instance, the metal-chelating properties of certain polyphenols (particularly catechins and procyanidins, abundant constituents of the *Annurca* apple) may directly sequester Hg^2+^ ions, reducing the availability of the metal for interference with calcium-regulatory systems (34). Additionally, restoration of redox homeostasis through a reduction in ROS generation may preserve the functional integrity of calcium channels and pumps involved in ion regulation (35).

Consistent with these observations, both immunocytochemistry and Western blot analyses confirmed that *Annurca* extracts attenuate the Hg-induced overexpression of PLSCR1, restoring its levels close to physiological conditions. This finding is critical because PLSCR1 upregulation is one of the main molecular mechanisms by which Hg promotes pathological PS exposure and consequently increases thrombotic risk. It is important to note that changes in PLSCR1 membrane expression and functional activation represent two distinct regulatory levels. In RBC, scramblase activity is primarily governed by intracellular Ca^2+^ availability; therefore, the reduction in phosphatidylserine exposure observed in the presence of *Annurca* extracts is mainly attributed to the inhibition of Ca^2+^-dependent PLSCR1 activation rather than to changes in protein abundance per se.

A key aspect emerging from this study is the lack of normalization of intracellular ATP levels and ATP11C flippase activity. Hg exposure caused a marked decrease in ATP levels and approximately 90% inhibition of ATP11C activity, which were not restored by *Annurca* treatment. This finding indicates that *Annurca* polyphenols do not act through energy-dependent pathways but rather modulate membrane enzymatic balance by regulating intracellular calcium and consequently PLSCR1 activity. This differentiates the mechanism of action of *Annurca* apple from that of other phenolic compounds previously investigated.

In particular, in our previous work we evaluated the protective effects of hydroxytyrosol (HT), the main phenolic metabolite of olive oil, on Hg-induced RBC damage (17, 18). Although both HT and *Annurca* extracts reduced Hg-induced PS exposure, important mechanistic differences emerged. Specifically, HT exhibited broader protection, associated not only with reduced PS exposure but also with restoration of ATP levels and ATP11C activity. HT acted through an energy/redox-dependent mechanism, restoring mitochondrial functionality and the flippase capacity to maintain membrane asymmetry (36). In contrast, *Annurca* extracts exerted a more selective effect, centered on calcium regulation and functional inhibition of PLSCR1. Taken together, these findings suggest that the two compounds share a common functional outcome, the reduction of PS exposure, but achieve it through complementary molecular pathways: HT primarily targets cellular bioenergetics, while *Annurca* acts on ionic homeostasis and prevents calcium-dependent activation of scramblases. This complementarity opens promising perspectives for combined nutritional strategies based on synergistic polyphenolic actions.

An often underestimated yet critical aspect of heavy metal toxicity is the role of uncontrolled intracellular calcium accumulation (37). While this study focused on calcium reduction as a primary protective mechanism, it is important to recognize that calcium overload is both a cause and a consequence of oxidative stress.

In RBC, Ca^2+^ overload may also activate additional pathways contributing to membrane remodeling, including calpain-mediated proteolysis and lipid peroxidation. Calpains are Ca^2+^-dependent cysteine proteases that can cleave cytoskeletal and membrane-associated proteins, thereby indirectly promoting phospholipid scrambling and membrane destabilization (38). Moreover, oxidative stress–driven lipid peroxidation may alter membrane fluidity and facilitate PS exposure. Although these mechanisms were not directly investigated in the present study, they represent complementary Ca^2+^-dependent processes potentially involved in Hg-induced RBC damage.

The ability of *Annurca* extracts to counteract calcium accumulation likely represents a multifunctional protective mechanism that not only regulates PLSCR1 but also prevents activation of calcium-dependent cell damage cascades. Future studies employing selective inhibitors of calcium-dependent proteases in combination with *Annurca* extracts may further elucidate this mechanism.

A particularly interesting finding is the differential protective effect observed between extracts from ripe and unripe fruits. Extracts from ripe fruits, both flesh and peel, consistently exhibited superior protection compared to those from unripe fruits. This suggests that post-harvest biological ripening induces significant changes in the *Annurca* polyphenolic profile. It is well established that during the traditional *Annurca* ripening process, conducted in open-air “melai” with natural sunlight exposure, substantial biochemical transformations occur, including chlorophyll degradation, carotenoid accumulation, and shifts in polyphenol composition (39). Ripening leads to a relative increase in specific polyphenols such as flavanols and procyanidins and to modifications in others, likely producing a profile with enhanced chelating and antioxidant properties particularly effective against Hg-induced calcium overload (20).

A critical consideration in interpreting these data concerns the translation from *in vitro* to *in vivo* conditions. The experiments were conducted using extract concentrations (0.3 and 1.5 μg CAEq/mL) and HgCl_2_ levels (20 μM) representing substantial cellular stress, albeit within the range of severe environmental exposure. *In vivo*, blood Hg concentrations in environmentally exposed individuals typically range from 0.25 to 5 μM, although tissue accumulation (e.g., RBC, kidneys, brain) can be considerably higher (40). The bioavailability of *Annurca* polyphenols remains a key issue not directly addressed in this study. Fruit-derived polyphenols undergo extensive intestinal and systemic metabolism, with only a fraction of the parent compounds reaching systemic circulation in unmetabolized form. Although specific data on *Annurca* polyphenol bioavailability in humans are lacking, it is reasonable to hypothesize that regular consumption of *Annurca* apples, given their procyanidin content up to 20-fold higher than conventional cultivars, could yield plasma polyphenol levels comparable to or even exceeding those observed with olive oil phenolics (41, 42).

This study provides a mechanistic rationale supporting the biological relevance of *Annurca* apple polyphenols in modulating RBC membrane alterations induced by Hg exposure. Given that *Annurca* is a geographically protected Campanian cultivar and a traditional component of the Mediterranean diet, its valorization as a polyphenol-rich food represents a valuable opportunity to explore diet-based strategies aimed at mitigating blood-related mechanisms associated with cardiovascular risk, rather than direct cardiovascular prevention.

It must be emphasized that the present study provides *in vitro* evidence obtained in an isolated human RBC system. Therefore, any extrapolation to clinical prevention strategies requires validation in more complex cellular models, animal studies evaluating systemic vascular and hemostatic endpoints, and ultimately randomized controlled clinical trials investigating the pharmacokinetics and pharmacodynamics of *Annurca* polyphenols in populations exposed to heavy metals.

In conclusion, the present study demonstrates that *Annurca* apple methanolic-polyphenolic extracts significantly attenuate Hg-induced RBC injury by reducing PS exposure through a calcium-dependent mechanism involving PLSCR1 modulation. Although this effect does not involve restoration of intracellular ATP levels, it results in a substantial reduction of membrane asymmetry disruption, a process mechanistically linked to procoagulant activity and thrombotic risk under conditions of environmental stress.

The mechanistic diversity observed in comparison with HT suggests that different classes of polyphenols may act through complementary molecular pathways, providing a biochemical basis for integrated nutritional approaches based on polyphenol-rich natural foods. Although the isolated RBC model does not recapitulate the full complexity of the cardiovascular system, it allows the identification of early blood-centered mechanisms, such as Ca^2+^-dependent procoagulant remodeling, that are relevant to cardiovascular risk. Future *in vivo* and *in silico* studies will be required to validate the systemic implications of these findings.

## Data Availability

The raw data supporting the conclusions of this article will be made available by the authors, without undue reservation.

## References

[1] Leventis PA Grinstein S. The distribution and function of phosphatidylserine in cellular membranes. Annu Rev Biophys. (2010) 39:407–27. doi: 10.1146/annurev.biophys.093008.131234 20192774

[2] Pabst G Keller S. Exploring membrane asymmetry and its effects on membrane proteins. Trends Biochem Sci. (2024) 49:333–45. doi: 10.1016/j.tibs.2024.01.007, 38355393

[3] Je V G T. Formation and function of phosphatidylserine and phosphatidylethanolamine in mammalian cells. Biochim Biophys Acta. (2013) 1831:543–54. doi: 10.1016/j.bbalip.2012.08.016 22960354

[4] Alghareeb SA Alfhili MA Fatima S. Molecular mechanisms and pathophysiological significance of eryptosis. Int J Mol Sci. (2023) 24:5079. doi: 10.3390/ijms24065079, 36982153 PMC10049269

[5] Repsold L Joubert AM. Eryptosis: an erythrocyte’s suicidal type of cell death. BioMed Res Int. (2018) 2018:9405617. doi: 10.1155/2018/9405617 29516014 PMC5817309

[6] Fens MHAM van Wijk R Andringa G van Rooijen KL Dijstelbloem HM Rasmussen TJ . A role for activated endothelial cells in red blood cell clearance: implications for vasopathology. Haematologica. (2012) 97:500–8. doi: 10.3324/haematol.2011.048694, 22102700 PMC3347679

[7] Notariale R Längst E Perrone P Crettaz D Prudent M Manna C. Effect of mercury on membrane proteins, anionic transport and cell morphology in human erythrocytes. Cell Physiol Biochem. (2022) 56:500–13. doi: 10.33594/000000572 36126286

[8] Perrone P Ortega-Luna R Manna C Álvarez-Ribelles Á Collado-Diaz V. Increased adhesiveness of blood cells induced by mercury chloride: protective effect of hydroxytyrosol. Antioxidants (Basel). (2024) 13:1576. doi: 10.3390/antiox13121576, 39765902 PMC11673208

[9] Zwaal RFA Comfurius P Bevers EM. Surface exposure of phosphatidylserine in pathological cells. Cell Mol Life Sci. (2005) 62:971–88. doi: 10.1007/s00018-005-4527-3, 15761668 PMC11924510

[10] Arashiki N Takakuwa Y Mohandas N Hale J Yoshida K Ogura H . ATP11C is a major flippase in human erythrocytes and its defect causes congenital hemolytic anemia. Haematologica. (2016) 101:559–65. doi: 10.3324/haematol.2016.142273, 26944472 PMC5004368

[11] Seki M Arashiki N Takakuwa Y Nitta K Nakamura F. Reduction in flippase activity contributes to surface presentation of phosphatidylserine in human senescent erythrocytes. J Cell Mol Med. (2020) 24:13991–4000. doi: 10.1111/jcmm.16010, 33103382 PMC7754070

[12] Kodigepalli KM Bowers K Sharp A Nanjundan M. Roles and regulation of phospholipid scramblases. FEBS Lett. (2015) 589:3–14. doi: 10.1016/j.febslet.2014.11.036, 25479087

[13] Nagata S Sakuragi T Segawa K. Flippase and scramblase for phosphatidylserine exposure. Curr Opin Immunol. (2020) 62:31–8. doi: 10.1016/j.coi.2019.11.009, 31837595

[14] Perrone P Notariale R Lettieri G Mele L La Pietra V Piscopo M . Protective effects of olive oil antioxidant phenols on mercury-induced phosphatidylserine externalization in erythrocyte membrane: insights into scramblase and flippase activity. Free Radic Biol Med. (2024) S0891-5849:01081–5. doi: 10.1016/j.freeradbiomed.2024.11.047 39613047

[15] Notariale R Moriello C Alessio N Del Vecchio V Mele L Perrone P . Protective effect of hydroxytyrosol against hyperglycemia-induced phosphatidylserine exposure in human erythrocytes: focus on dysregulation of calcium homeostasis and redox balance. Redox Biol. (2025) 85:103783. doi: 10.1016/j.redox.2025.103783, 40700934 PMC12304697

[16] Darabi M Kontush A. Phosphatidylserine in atherosclerosis. Curr Opin Lipidol. (2016) 27:414–20. doi: 10.1097/MOL.0000000000000298, 27070078

[17] Perrone P Spinelli S Mantegna G Notariale R Straface E Caruso D . Mercury chloride affects band 3 protein-mediated anionic transport in red blood cells: role of oxidative stress and protective effect of olive oil polyphenols. Cells. (2023) 12:424. doi: 10.3390/cells12030424, 36766766 PMC9913727

[18] Notariale R Perrone P Mele L Lettieri G Piscopo M Manna C. Olive oil phenols prevent mercury-induced phosphatidylserine exposure and morphological changes in human erythrocytes regardless of their different scavenging activity. Int J Mol Sci. (2022) 23:5693. doi: 10.3390/ijms23105693, 35628502 PMC9147954

[19] Ahmad S Mahmood R. Mercury chloride toxicity in human erythrocytes: enhanced generation of ROS and RNS, hemoglobin oxidation, impaired antioxidant power, and inhibition of plasma membrane redox system. Environ Sci Pollut Res Int. (2019) 26:5645–57. doi: 10.1007/s11356-018-04062-5, 30612358

[20] Perrone P Moriello C Alessio N Manna C D’Angelo S. Cytoprotective potential of *Annurca* apple polyphenols on mercury-induced oxidative stress in human erythrocytes. Int J Mol Sci. (2025) 26:8826. doi: 10.3390/ijms26188826, 41009393 PMC12469998

[21] Perrone P Palmieri S Piscopo M Lettieri G Eugelio F Fanti F . Antioxidant activity of *Annurca* apple by-products at different ripening stages: a sustainable valorization approach. Antioxidants. (2025) 14:941. doi: 10.3390/antiox14080941, 40867836 PMC12382625

[22] Męczarska K Cyboran-Mikołajczyk S Solarska-Ściuk K Oszmiański J Siejak K Bonarska-Kujawa D. Protective effect of field horsetail polyphenolic extract on erythrocytes and their membranes. Int J Mol Sci. (2025) 26:3213. doi: 10.3390/ijms26073213, 40244071 PMC11989917

[23] Fratianni F De Giulio A Sada A Nazzaro F. Biochemical characteristics and biological properties of *Annurca* apple cider. J Med Food. (2012) 15:18–23. doi: 10.1089/jmf.2011.0042 21861727

[24] Goldsborough E Osuji N Blaha MJ. Assessment of cardiovascular disease risk: a 2022 update. Endocrinol Metab Clin N Am. (2022) 51:483–509. doi: 10.1016/j.ecl.2022.02.005, 35963625

[25] Reis AH. Towards a new understanding of the molecular mechanisms of cardiovascular disease. Discov Med. (2019) 28:189–94. 31928626

[26] Jebari-Benslaiman S Larrea-Sebal A Benito-Vicente A Martín C. Cardiovascular disease, atherosclerosis and familial hypercholesterolemia: from molecular mechanisms causing pathogenicity to new therapeutic approaches. Int J Mol Sci. (2023) 24:7659. doi: 10.3390/ijms24087659, 37108820 PMC10145605

[27] Aschner M Carvalho C. The biochemistry of mercury toxicity. Biochim Biophys Acta Gen Subj. (2019) 1863:129412. doi: 10.1016/j.bbagen.2019.129412, 31401179

[28] Kobal AB Horvat M Prezelj M Briski AS Krsnik M Dizdarevic T . The impact of long-term past exposure to elemental mercury on antioxidative capacity and lipid peroxidation in mercury miners. J Trace Elem Med Biol. (2004) 17:261–74. doi: 10.1016/S0946-672X(04)80028-2, 15139389

[29] Dos Santos Chemelo V Bittencourt LO Aragão WAB Dos Santos SM Souza-Rodrigues RD Ribeiro CHMA . Long-term exposure to inorganic mercury leads to oxidative stress in peripheral blood of adult rats. Biol Trace Elem Res. (2021) 199:2992–3000. doi: 10.1007/s12011-020-02411-5, 32997227

[30] Moriello C De Rosa C D’Angelo S Pasquale P. Polyphenols and chronic myeloid leukemia: emerging therapeutic opportunities. Hema. (2025) 6:28. doi: 10.3390/hemato6030028

[31] Perrone P D’Angelo S. Hormesis and health: molecular mechanisms and the key role of polyphenols. Food Chem Adv. (2025) 7:101030. doi: 10.1016/j.focha.2025.101030

[32] Closse C Dachary-Prigent J Boisseau MR. Phosphatidylserine-related adhesion of human erythrocytes to vascular endothelium. Br J Haematol. (1999) 107:300–2. doi: 10.1046/j.1365-2141.1999.01718.x 10583215

[33] Yeh J-H Chung H-M Ho C-M Jan C-R. Mercury-induced Ca2+ increase and cytotoxicity in renal tubular cells. Life Sci. (2004) 74:2075–83. doi: 10.1016/j.lfs.2003.09.046, 14967201

[34] Lakey-Beitia J Burillo AM La Penna G Hegde ML Rao KS. Polyphenols as potential metal chelation compounds against Alzheimer’s disease. J Alzheimer’s Dis. (2021) 82:S335–57. doi: 10.3233/JAD-200185, 32568200 PMC7809605

[35] Perrone P D’Angelo S. Gut microbiota modulation through Mediterranean diet foods: implications for human health. Nutrients. (2025) 17:948. doi: 10.3390/nu17060948, 40289944 PMC11944315

[36] Noguera-Navarro C Montoro-García S Orenes-Piñero E. Hydroxytyrosol: its role in the prevention of cardiovascular diseases. Heliyon. (2023) 9:e12963. doi: 10.1016/j.heliyon.2023.e12963, 36704293 PMC9871206

[37] Marchetti C. Role of calcium channels in heavy metal toxicity. ISRN Toxicol. (2013) 2013:184360. doi: 10.1155/2013/184360, 23724297 PMC3658387

[38] Vanderklish PW Bahr BA. The pathogenic activation of calpain: a marker and mediator of cellular toxicity and disease states. Int J Exp Pathol. (2000) 81:323–39. doi: 10.1111/j.1365-2613.2000.00169.x, 11168679 PMC2517738

[39] D’Angelo S Cimmino A Raimo M Salvatore A Zappia V Galletti P. Effect of reddening-ripening on the antioxidant activity of polyphenol extracts from cv. “*Annurca*” apple fruits. J Agric Food Chem. (2007) 55:9977–85. doi: 10.1021/jf071773a, 17960886

[40] Eom S-Y Choi S-H Ahn S-J Kim D-K Kim D-W Lim J-A . Reference levels of blood mercury and association with metabolic syndrome in Korean adults. Int Arch Occup Environ Health. (2014) 87:501–13. doi: 10.1007/s00420-013-0891-8, 23824410

[41] Kaeswurm JAH Burandt MR Mayer PS Straub LV Buchweitz M. Bioaccessibility of apple polyphenols from peel and flesh during oral digestion. J Agric Food Chem. (2022) 70:4407–17. doi: 10.1021/acs.jafc.1c08130, 35357186 PMC9012181

[42] Tenore GC Campiglia P Ritieni A Novellino E. In vitro bioaccessibility, bioavailability and plasma protein interaction of polyphenols from *Annurca* apple (*M. pumila* miller cv *Annurca*). Food Chem. (2013) 141:3519–24. doi: 10.1016/j.foodchem.2013.06.051, 23993515

